# Materia Medica, or Pharmacology and Therapeutics

**Published:** 1853-01

**Authors:** William Tully


*Materia Medica, or Pharmacology and Therapeutics.* By William Tully, M. D. Vol. I, No. 1. Nov., 1852.

This is the first of a series of numbers (64 pages each No.) proposed to be issued monthly by Drs. Church and Seegar, Springfield, Mass., for an indefinate period, till the manuscript lectures of Dr. Tully are exhausted. A circular, setting forth the plan of the enterprise, and its importance, was published in a former number of this Journal.

By the warm recommendations which the announcement of the undertaking called forth from distinguished members of the profession, our anticipations had been highly raised, and we proceeded to examine the first No. of the series with eagerness. The paragraph, however, which first met our eye on opening the work, dissipated all our expectations. The paragraph alluded to was as follows: "As appears to me, there is sufficient evidence that those remedial agents which are taken into the alimentary canal, make all their medicinal impression upon the nerves of its inner parietes, whence it is propagated by some one of the modes of sympathy already explained, to the part or parts in which we perceive the first manifestations of their operations; — that no part or portion of one medicine in fifty, or a hundred, ever leaves the alimentary canal, except to pass off with the fæces; and that, when some part or portion of a medicine happens to be capable of being taken into the mass of the circulating fluids, it produces all its medicinal effects before it is so taken up; and no medicinal effect (at least as a very general rule) after it leaves the alimentary canal."

On looking over the No. we find that a greater part of it is taken up with a discussion of the proposition contained in the quotation just made.

The author, thus, adopts as the basis of the materia medica, the obsolete dogma of *exclusive solidism.* With this vicious principle pervading it, all the learning or thought which the work may contain, cannot save it from becoming a monument of the past errors of science, instead of an exponent of our present knowledge. Such a work, at this day, will only prove a failure, bringing pecuniary loss and mortification to those engaged in the undertaking. The pernicious doctrine upon which it rests is too thoroughly exploded, for its revival to be attended by any danger of mischief. It is greatly to be regretted that the friends of the venerable Dr. Tully have encouraged this project; and the most friendly wish, with regard to it, which can be expressed is, that the inadequate demand for the first number may lead to a speedy abandonment of the publication.

